# Biomarkers of Eating Disorders Using Support Vector Machine Analysis of Structural Neuroimaging Data: Preliminary Results

**DOI:** 10.1155/2015/924814

**Published:** 2015-11-18

**Authors:** Antonio Cerasa, Isabella Castiglioni, Christian Salvatore, Angela Funaro, Iolanda Martino, Stefania Alfano, Giulia Donzuso, Paolo Perrotta, Maria Cecilia Gioia, Maria Carla Gilardi, Aldo Quattrone

**Affiliations:** ^1^IBFM-CNR, 88100 Catanzaro, Italy; ^2^IBFM-CNR, University of Milan-Bicocca, H S. Raffaele, Via Fratelli Cervi 93, 20090 Segrate, Italy; ^3^Associazione Centro Trauma Ippocampo, Via Rossini 5, 87100 Castrolibero, Italy; ^4^Unit of Neurology, “Magna Graecia” University, 88100 Catanzaro, Italy

## Abstract

Presently, there are no valid biomarkers to identify individuals with eating disorders (ED). The aim of this work was to assess the feasibility of a machine learning method for extracting reliable neuroimaging features allowing individual categorization of patients with ED. Support Vector Machine (SVM) technique, combined with a pattern recognition method, was employed utilizing structural magnetic resonance images. Seventeen females with ED (six with diagnosis of anorexia nervosa and 11 with bulimia nervosa) were compared against 17 body mass index-matched healthy controls (HC). Machine learning allowed individual diagnosis of ED versus HC with an Accuracy ≥ 0.80. Voxel-based pattern recognition analysis demonstrated that voxels influencing the classification Accuracy involved the occipital cortex, the posterior cerebellar lobule, precuneus, sensorimotor/premotor cortices, and the medial prefrontal cortex, all critical regions known to be strongly involved in the pathophysiological mechanisms of ED. Although these findings should be considered preliminary given the small size investigated, SVM analysis highlights the role of well-known brain regions as possible biomarkers to distinguish ED from HC at an individual level, thus encouraging the translational implementation of this new multivariate approach in the clinical practice.

## 1. Introduction

Eating disorders (ED) are typically adolescent-onset psychiatric conditions that cause serious disturbances to everyday diet, such as eating extremely small amounts of food or severely overeating. Female gender has been demonstrated as a potent risk factor for eating disorders [1], but how much this association can be attributed to biological rather than social factors is uncertain [2]. The most investigated clinical phenotypes of ED are anorexia nervosa (AN) and bulimia nervosa (BN). AN is a serious mental disorder that leads to death in approximately 10% of cases [3]. According to the new DSM-5 criteria, to be diagnosed as having AN a person must display (a) persistent restriction of energy intake relative to requirements leading to a significantly low body weight; (b) intense fear of gaining weight or becoming fat, even though they are underweight; and (c) disturbance in the way in which one's body weight or shape is experienced, undue influence of body weight or shape on self-evaluation, or denial of the seriousness of the current low body weight. Otherwise, BN is characterized by frequent episodes of binge eating followed by inappropriate behaviors such as self-induced vomiting to avoid weight gain. DSM-V criteria reduce the frequency of binge eating and compensatory behaviors that people with BN must exhibit, to once a week from twice weekly as specified in DSM-IV.

To date, individual diagnosis of ED is based only on a clinical interview complemented by physical, psychopathological, and behavioral examinations aimed at assessing the existence of physical, emotional, behavioral and cognitive disturbances. However, ED diagnosis is unstable, with clinical features changing over time (i.e., weight normalization [11]) and often switching from anorexia to bulimia [4]. For this reason, there is an urgent need to identify biomarkers which may be used for helping and improving early diagnosis, treatment planning, and monitoring of disease progression. In the past 10 years, considerable effort has been expended in developing advanced neuroimaging methods. As a result, a plethora of functional and structural neuroimaging studies have been performed to unravel the pathophysiological mechanisms of ED [5–9]. Whereas the vast majority of these studies reported in AN patients global reductions of total gray and white matter [10], as well as cortical thickness [11], a number of recent studies have emphasized regional group differences. What has been proposed was that AN patients are characterized by widespread brain abnormalities involving (a) the mesolimbic regions (striatum, hippocampus, amygdala, and cerebellum), (b) the dorsolateral prefrontal cortex, (c) the visual cortex, and (d) the cerebellum [12]. Otherwise, neuroimaging literature about BN summarizes the presence of specific involvement of the reward neural system (ventral striatum, nucleus caudate, anterior cingulate cortex (ACC), orbitofrontal cortex (OFC)), hypothesizing that during binge eating a person must consume greater quantities of food to achieve the feeling of satisfaction, like an addict [13–15].

Although significant results have been achieved, the disadvantage of these studies is that they reported neurobiological abnormalities comparing patients and controls at a group level, with consequently limited clinical translation at the individual level. For this reason, attention has recently turned toward alternative kinds of analyses of neuroimaging data. In the last few years, there has been growing interest within the neuroimaging community in classification methods, including machine learning methods. These techniques are based on algorithms able to automatically extract multiple pieces of information from image sets without requiring* a-priori* hypotheses of where they may be found on images. The aim of these methods is to maximize the distance between image groups in order to classify individual structural or functional brain images. Several studies have assessed the clinical relevance of these techniques showing very promising findings mainly in the neurological realm. For instance, machine learning techniques are able to identify very reliable imaging biomarkers allowing individual diagnoses of Alzheimer's disease [16, 17], Mild Cognitive Impairment [18], and Parkinson's disease [19, 20] with an Accuracy of above 90%. In the psychiatric realm, this kind of advanced neuroimaging method is in its relative infancy. Although some interesting applications have been made in patients with posttraumatic stress disorders [21], depression disorders [22], and first-episode psychosis [23], there are no studies investigating the potential role of these methods in ED.

For this reason, this study was aimed at employing a validated supervised machine learning method to define reliable neuroimaging biomarkers useful to distinguish individual with diagnosis of ED patients from healthy controls (HC) by means of structural T1-weighted magnetic resonance images (MRIs). This method makes uses of Principal Components Analysis (PCA) in order to extract the most informative features from MR images [24], while the Support Vector Machine (SVM) approach was used to perform classification [25]. Maps of voxel-based pattern distribution of structural brain differences were generated. These maps show the significance of each image voxel for SVM group discrimination.

## 2. Methods

### 2.1. Participants

From 2011 to 2012, a total of 103 patients presenting a first diagnosis of ED were enrolled in this study. All patients were diagnosed by two psychiatrists specialized in ED using the Structured Clinical Interview for Diagnosis (SCID) for DSM-IV-TR. After reviewing the diagnostic information, the psychiatrists made a final diagnosis of ED subtype and proposed the patient's participation in this research project. Inclusion criteria were as follows: (1) age range from 18 to 40 years, (2) being female, and (3) right-handedness. Exclusion criteria were as follows: (1) neurological illness (such as Epilepsy or mental retardation); (2) Axis II disorders (using the SCID-II for DSM-IV-TR) to exclude comorbidity with personality disorders; (3) presence of brain lesions as well as history of cerebrovascular disease, head trauma, or hypertension; (4) psychotropic medication; (5) drug or alcohol abuse; (6) claustrophobia; and (7) past recovery from ED symptoms or psychiatric disorders. After a careful evaluation of these criteria, 17 females with ED were eligible for this study. This group included 11 patients fulfilling DSM-IV criteria for BN and six patients fulfilled DSM-IV criteria for AN restrictive-type. Duration of illness was rather short for all patients (mean duration: 16 ± 5 months).

ED patients were compared with a group of HC. Eighty-one healthy volunteers were recruited by local advertisements. Inclusion criteria for the HC recruitment were as follows: (1) no previous histories of neurological or psychiatric diseases or abnormal brain MRIs and (2) being within the normal range on the Italian version of Minnesota Multiphasic Personality Inventory-2 (MMPI-2) [26]. From this large group, we only enrolled subjects having similar demographical characteristics of those detected in ED patients. Particular attention was paid to potential confounding factors, such as BMI, previously demonstrated to influence brain anatomy [27]. Thus, ED and HC individuals were individually pair-matched by a computer-generated program, according to their age, educational level, and BMI (±2) (for further information, see Supplementary Materials available online at http://dx.doi.org/10.1155/2015/924814). A total sample of seventeen female HC was then enrolled in this study.

All participants gave written informed consent to participate in the present study, approved by the Local Ethical Committee according to the Declaration of Helsinki.

#### 2.1.1. Psychiatric Assessment

Before entering the study, participants completed a battery of self-evaluation questionnaires that included the following.

*Eating Disorders Inventory-2 (EDI-2)*. It is a worldwide validated questionnaire that provides a multidimensional evaluation of the psychological characteristics of AN and BN [28].

*Traumatic Experiences Checklist (TEC)*. It is a self-report measure addressing potentially traumatizing events [29]. Different scores can be calculated including a cumulative score and scores for emotional neglect, emotional abuse, physical abuse, sexual harassment, sexual abuse, and bodily threat from a person.

*Dissociative Experiences Scale v. II (DES-II)*. It is a lifetime 28-item, self-rating questionnaire developed specifically as a screening instrument to identify subjects that are likely to have dissociative symptoms [30].

*Somatoform Dissociation Questionnaire-20 (SDQ-20)*. It is a self-rating scale developed to the investigated somatic component of dissociation. The SDQ-20 discriminates between dissociative and affective disorders (mood and anxiety disorders) and psychotic symptoms, but a cut-off score is not available [31].

*Parental Bonding Instrument (PBI)*. Perceived parental rearing styles were assessed using the Italian version of PBI. PBI is a self-reporting scale with 25 items to rate paternal or maternal attitude during the first 16 years and has four items comprising care and overprotection factors [32].

*Eating Attitude Test-26 (EAT-26)*. It is a 26-item self-rated questionnaire for evaluating ED-related symptoms [33]. The results are presented as a total score (range, 0–78).

*Body Image Dimensional Assessment (BIDA)*. The BIDA is a silhouette-based scale that starts from neutral figural stimuli and attributes a direct quantitative value to the subject's own current and ideal body image, the most sexually attractive figure, and the most common figure of same-gender-and-age fellows [34].

Finally, for assessing anxiety symptoms, we employed the Hamilton rating scale for anxiety (HAM-A), whereas for defining depression status we employed the Beck Depression Inventory (BDI).

#### 2.1.2. MRI Acquisition

Brain MRI was performed according to our routine protocol by a 3 T scanner with an 8-channel head coil (Discovery MR-750, GE, Milwaukee, WI, USA). Structural MRI data were acquired using a 3D T1-weighted spoiled gradient echo sequence with the following parameters: TR: 9.2 ms, TE: 3.7 ms, flip angle 12°, and voxel-size 1 × 1 × 1 mm^3^. Subjects were positioned to lie comfortably in the scanner with a forehead-restraining strap and various foam pads to ensure head fixation. All acquired images were visually inspected by expert physicians and neuroradiologists to ensure that none showed signal artifacts.

### 2.2. Classification of MRI Studies: The Machine Learning Method

We employed a validated supervised machine learning method [20] for the individual differential diagnosis of ED. PCA was applied to whole-brain structural T1-weighted MRIs in order to extract the most informative features for class discrimination, while a SVM algorithm [25] was used to perform classification.

#### 2.2.1. Image Preprocessing

Using the “Tools For NIfTI And ANALYZE Image” toolbox (http://www.mathworks.com/matlabcentral/fileexchange/8797), original images were imported into the Matlab platform (Matlab version R2011b, The MathWorks, Natick, MA).

Image preprocessing was achieved by means of the VBM8 toolbox [35] implemented in the SPM8 software package [36]. This step involved (1) reorientation; (2) cropping; (3) skull-stripping; (4) spatial nonlinear normalization to the MNI152 reference space; (5) smoothing using a Gaussian kernel with full-width at half maximum of 8 × 8 × 8 mm. Resulting nonmodulated whole-brain images were used as input to the feature extraction procedure. Final volume size was of 121 × 145 × 121 voxels. VBM8 was also employed to automatically calculate the total gray matter (GM) and white matter (WM), as well as cerebrospinal fluid (CSF) volumes.

It is worth noting that all images were visually controlled after each step of the preprocessing flow in order to identify possible problems occurring as a consequence of the applied operations.

#### 2.2.2. Feature Extraction

After preprocessing, PCA was applied to structural T1-weighted MRIs considering whole brain, in order to select the most informative features for class discrimination [24, 37]. PCA mainly consists of two steps: the first step is the application of an orthogonal transformation to the dataset, which results in a set of values of linearly uncorrelated variables, or eigenvectors, called “principal components”; extracted principal components are ordered by their variance. The maximum number of eigenvectors that can be extracted with a nonzero associated eigenvalue is related to the lower sample dimension of the dataset. In this case, the number of extracted eigenvectors with a nonzero associated eigenvalue can at most be equal to *N* − 1, *N* being the number of subjects involved.

The second step is the projection of the dataset itself into the PCA subspace, which heavily decreases the number of features to be handled. Features resulting from this analysis are called PCA coefficients, and they are the ones used for classification in place of the original dataset [38]. For group comparison, we also studied the percentage of retained variance as a function of the number of considered principal components.

Obtained PCA coefficients were finally ordered according to their Fisher Discriminant Ratio (FDR), with the aim of identifying the most discriminative PCA coefficients. Indeed, FDR provides information about the class discriminatory power of a given component, that is, the ability of each component to separate the samples belonging to the two classes. FDR was calculated as follows:

(1) $$\begin{array}{c} \text{FDR}=\frac{{\left(\mu_{1}-\mu_{2}\right)}^{2}}{\sigma_{1}^{2}+\sigma_{2}^{2}}, \end{array}$$

*μ*_*i*_ and *σ*_*i*_^2^ being the mean and the variance of the *i*th class, respectively.

#### 2.2.3. Classification Algorithm

SVM algorithm was used to perform classification [20, 25, 39]. Given a set of training data, each piece consisting of an input vector *x*_*i*_ *R* ^*N*^ (where *i* runs from 1 to the number *N* of samples) and the corresponding label *t*_*i*_  {±1}, the task of the SVM is then to compute the optimal separating hyperplane between the two training classes that will be able to classify unseen examples (*x*, *t*) in a correct way. This is done in terms of distance between classes; that is, the optimal separating hyperplane is computed so that its distance from the two training classes to be divided is maximized. The optimal separating hyperplane will then be used as a decision function to classify unseen data as belonging to one of the two training classes. Mathematically, the decision function is defined as follows:

(2) $$\begin{array}{c} y\left(\;x\;\right)=\overset{N}{\underset{n=1}{\sum }}a_{n}\cdot t_{n}\cdot f\left(\;x,x_{n}\;\right)+b. \end{array}$$

*N* is the number of samples belonging to the training set; *a*_*n*_ is a weight constant; *f*(*x*, *x*_*n*_) is the kernel function; *b* is a threshold parameter. Using this decision function, class *y*(*x*) for unseen data (*x*, *t*) can be predicted.

The implementation of the SVM classification algorithm was carried out using the biolearning toolbox in Matlab. Original datasets were divided into two discrimination groups: ED versus HC. The extracted PCA coefficients and the corresponding labels were used as features to train the classification algorithm [20]. A number *k* of PCA coefficients was used, where *k* runs from 1 to the total number *N*_PC_ of extracted PCA coefficients. A linear kernel was chosen for two reasons: (1) it is able to improve generalization ability; (2) it is the only kernel function that allows the computation of weights and, thus, the generation of voxel-based pattern distribution maps of brain structural differences.

#### 2.2.4. Performances of the Classifier

In order to evaluate the performance of the supervised machine learning method, subjects were randomly divided into 20 subsets, each one containing the same proportion of class labels. Evaluation was performed* via* 20-fold Cross-Validation (CV), by which in turn the training of the classifier was performed using 19 subsets and the testing was performed using the remaining one. This procedure was then repeated 20 times, until all subsets were used once as testing set. In addition, classification performance was also evaluated by 10-fold CV.

Accuracy, Specificity, and Sensitivity were computed over the first *k* PCA coefficients, where *k* runs from 1 to the total number *N*_PC_ of extracted PCA coefficients, as follows:

(3) Accuracyi=NCCN,Specificityi=ACCACC+BIC,Sensitivityi=BCCBCC+AIC,

where *N* is the total number of images which underwent classification; *N*_CC_ is the total number of Correctly Classified (CC) images; *A*_CC_ is the number of CC images belonging to the first group; *A*_IC_ is the number of Incorrectly Classified (IC) images belonging to the first group; *B*_CC_ is the number of CC images belonging to the second group; *B*_IC_ is the number of IC images belonging to the second group. It is worth noting that for each round of CV, image preprocessing and feature extraction were performed separately on the training and the testing sets. Accuracy was evaluated as a function of the number of employed PCA coefficients.

#### 2.2.5. Voxel-Based Pattern Distribution

For each discrimination group, maps of voxel-based pattern distribution of brain structural differences were generated. These maps show how significant each image voxel is for SVM group discrimination [20]. In the training phase, in fact, SVM assigns a specific weight to each sample (i.e., the vector of extracted PCA coefficients of each subject) in the training set, this weight reflecting the importance of that sample for group discrimination. In our case, deriving discriminative voxels basing on the SVM weights cannot be done in a direct way, because we use PCA coefficients as input to the SVM instead of image voxels. In order to do this, an intermediate step was needed, that is, back-projection of each sample (i.e., PCA coefficients) from the PCA space to the voxel space. Through this operation, we obtained a back-projected image of the brain of each subject in the voxel space. Finally, maps of values showing the importance of each voxel for group discrimination based on the SVM weights were then obtained by multiplying each back-projected brain of the training set with the corresponding weight assigned by SVM and by summing the results on a voxel basis [16, 20].

However, the multivariate SVM algorithm was not designed to provide single features and their importance. As a consequence, the method to derive discriminative features from the SVM model is a tweak that should be used with caution, because the interpretation of weights assigned by SVM during the training phase could lead to incorrect conclusions. In order to avoid this, we applied the method proposed by Haufe and colleagues to compute activation patterns for backward models [40]. This method ensures the correct interpretation of weights assigned by SVM. Accordingly, in addition to the weight map, we obtained a map of voxel-based pattern distribution of MR image differences between ED and HC.

Both the weight map and the voxel-based pattern distribution obtained using the method proposed by Haufe and colleagues [40] were normalized to a range between 0 and 1, expressed by a proper color scale and superimposed on a standard stereotactic brain for spatial localization. This approach allowed the identification of new MR-related biomarkers for the diagnosis of ED patients (see Supplementary Materials for further information).

#### 2.2.6. Statistical Analysis

Statistical analysis was performed with STATISTICA Version 6.0 (http://www.statsoft.com/). Assumptions for normality were tested for all continuous variables by using the Kolmogorov-Smirnov test. All variables were normally distributed, except for educational level. Then, Unpaired *t*-test and Mann-Whitney *U* test were applied appropriately to assess potential differences between groups for all demographic clinical and MRI variables. All statistical analyses had a 2-tailed alpha level of <0.05 for defining significance.

## 3. Results

### 3.1. Clinical Data

Compared with age-/sex-/BMI-matched controls, ED patients did not show global anatomical atrophies in white or gray matter brain volumetry. At a behavioral level, ED group displayed a well-known psychopathological profile (Table 1 and Supplementary Materials). In particular, EDI-2 demonstrated that ED patients had higher scores for (a) drive for thinness scale (*t* = 4.45; *p*-level < 0.00001); (b) bulimia scale (*t* = 2.69; *p*-level = 0.01); (c) interoceptive awareness scale (*t* = 3.81; *p*-level = 0.0006); (d) asceticism scale (*t* = 3.81; *p*-level = 0.0006); (e) body dissatisfaction (*t* = 3.5; *p*-level = 0.001); (f) interpersonal distrust scale (*t* = 2.07; *p*-level = 0.04); and (g) impulse regulation scale (*t* = 2.46; *p*-level = 0.02). Otherwise, no significant differences were detected for Perfectionism, Ineffectiveness, Maturity Fears, and Social Insecurity scales, in agreement with previous studies [13].

### 3.2. The Machine Learning Method

**Figure 1:**
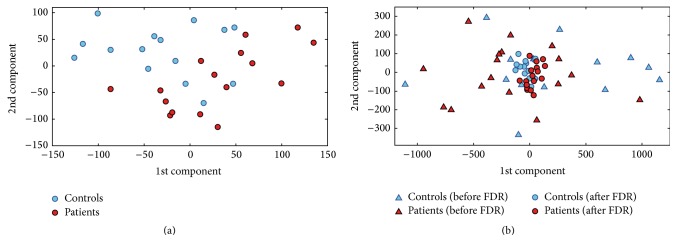
Plot of the PCA coefficients that showed the highest FDR (a) and joint plot of the PCA coefficients before (triangles) and after (circles) FDR ranking (b) for the ED versus HC group discrimination (1st and 2nd components). Data from a single round of CV are shown as a representative example.

Among MR images acquired for this study, no images were excluded from the subsequent analysis due to problems with image quality or problems occurred during preprocessing. As a representative example, 1st and 2nd extracted PCA coefficients that showed the highest FDR are plotted in Figure 1(a) for the ED versus HC group discrimination (data from a single round of CV). In this case, the number of subject involved was equal to 31 (16 ED, 15 HC). The total number of extracted PCA coefficients was equal to 30. The analysis of variance for the ED versus HC group discrimination showed that the percentage of variance retained by the first principal component was equal to 27.0%, while the number of extracted principal components accounting for 50% and 95% of the whole variance was 6 and 27, respectively.

Table 2 shows FDR values of the 30 features (PCA coefficients) used for the ED versus HC group discrimination. Data from a single round of CV are shown as a representative example. As it can be seen, in this case the 8th PCA coefficient showed the highest FDR value, thus resulting the most important feature for group discrimination.

In Figure 1(b), 1st and 2nd extracted PCA coefficients that showed the highest FDR (i.e., after FDR raking) are plotted jointly with 1st and 2nd extracted PCA coefficients (before FDR ranking). As it is shown in this plot, FDR allows finding those features for which discrimination between groups is maximized.

### 3.3. Classification Algorithm

**Figure 2:**
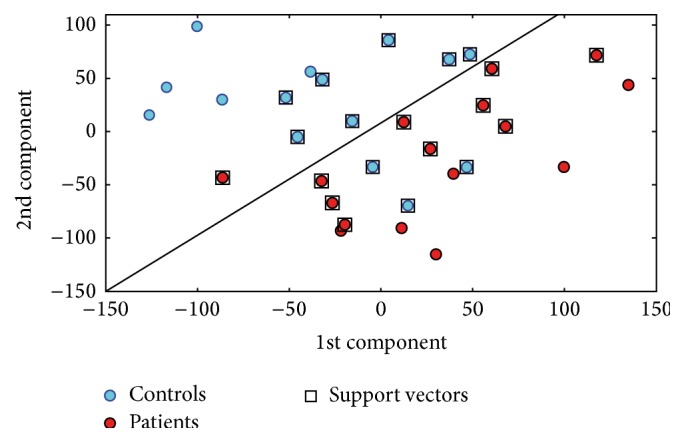
Decision function for the ED versus HC group discrimination (1st and 2nd components with highest FDR). Data from a single round of CV are shown as a representative example.

Figure 2 shows the decision function resulting from the SVM training phase for the ED versus HC group discrimination (1st and 2nd components with highest FDR).

### 3.4. Performances of the Classifier

When considering 20-fold CV approach, Accuracy, Specificity and Sensitivity of the classifier for ED versus HC group discrimination were calculated over a number of PCA coefficients ranging from 1 to 32. When using 31 PCA coefficients, Accuracy, Specificity and Sensitivity reached their best values of 0.85, 0.73 and 0.93, respectively.

**Figure 3:**
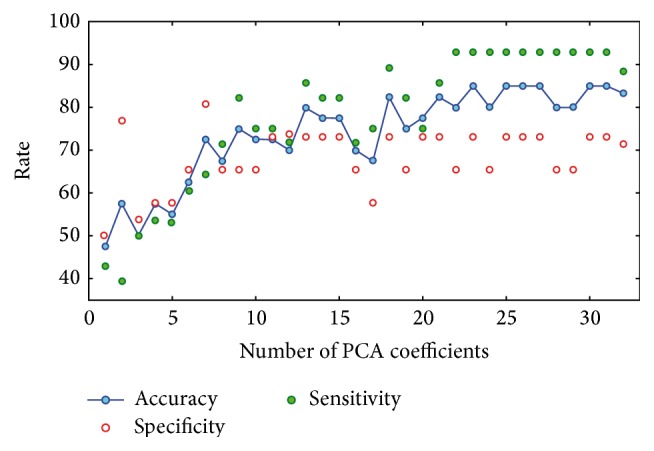
Accuracy, Specificity, and Sensitivity of classification as a function of the number of employed PCA coefficients for the ED versus HC group discrimination (20-fold CV).

Figure 3 shows Accuracy, Specificity and Sensitivity as a function of the number of employed PCA coefficients for the ED versus HC group discrimination. As expected, the performance of the classification algorithm increases with the number of employed PCA coefficients.

When considering 10-fold CV approach, Accuracy, Specificity and Sensitivity of the classifier for ED versus HC group discrimination were calculated over a number of PCA coefficients ranging from 1 to 30. In this case, when using 21 PCA coefficients, Accuracy, Specificity and Sensitivity reached their best values of 0.80, 0.72 and 0.96, respectively.

### 3.5. Voxel-Based Pattern Distribution

**Figure 4:**
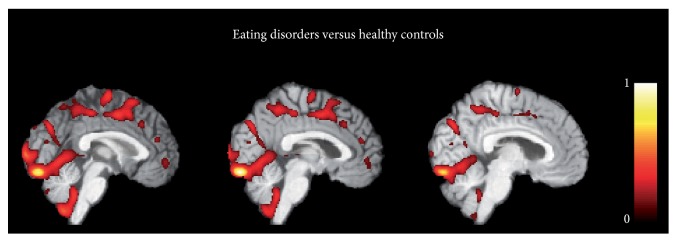
Voxel-based pattern distribution map of brain structural differences between ED patients and healthy controls (sagittal view, threshold = 50%). Voxel-based pattern distribution (normalized to a range between 0 and 1) is expressed according to the color scale and superimposed on a standard stereotactic brain for spatial localization.

Figure 4 shows the voxel-based pattern distribution map of brain structural differences between ED patients and HC. The pattern of differences emerged mainly in the occipital cortex and the posterior cerebellar lobule. Moreover, other brain regions involved in regulation of emotional processing known to be damaged in ED patients were detected: precuneus, sensorimotor and premotor cortices as well as the ACC and OFC.

## 4. Discussion

The pathophysiological mechanisms underlying ED remain a matter of debate. In the last few years, several meta-analyses have tried to summarize the large amount of evidence coming from behavioral and neuroimaging realms, providing different key of lectures. At a behavioral level, taking into account the clinical heterogeneity of ED subtypes, a large amount of literature highlights the AN-related psychopathology characterized by excessive Perfectionism, cognitive-behavioral rigidity, asceticism, ruminations, obsessions about food and excessive concerns about weight and shape, whereas BN patients would seem to be characterized by an impulsivity trait with a combination of heightened sensitivity to reward and impaired inhibitory control [15, 41, 42]. As concerns neuroimaging findings, although important pathological markers have been found describing neurobiological differences between AN and BN subtypes, the majority of these findings has never been translated into clinical practice. For this reason, the implementation of supervised whole-brain automatic classification methods may become an essential step for improving clinical management of psychiatric patients in longitudinal and prospective studies [43]. SVM has been proposed as a new approach for identifying sensitive biomarkers (or combinations of them) that allow for automatic discrimination of individual subjects. In this work we proposed, for the first time, a SVM algorithm that, working on structural neuroimaging data at a whole-brain level, reached an optimal individual classification in the comparisons between ED patients with controls. The strengths of this work were: (a) the detected pattern of neural abnormalities that allowed the SVM approach to reach this great Accuracy involved well-known brain regions strongly involved in the pathophysiological mechanisms of ED [5–9, 12]; (b) the classification Accuracy in the discrimination of all individual ED patients with respect to controls was equal or higher than those detected in previous studies employing machine learning to classify other psychiatric disorders: ~80–85% in schizophrenic patients [21], 81% in depression disorders [22] and ~75% in first-episode psychosis [23]; (c) the employment of HC matched for BMI, a critical variable known to influence brain anatomy [27] and sparsely controlled in other neuroimaging studies investigating ED patients.

Pattern recognition analysis used to classify ED patients from HC depicted mainly the involvement of the: (a) cerebellum, (b) reward-related cortical regions, (c) occipital cortex and (d) sensorimotor cortex. (a) The cerebellum is a multidimensional brain region involved in a plethora of motor, cognitive and emotional functions. Recent studies have also highlighted the role of the cerebellum in visceral and autonomic regulation, specifically the cerebellar vermis, which has a role in feeding behavior and appetite regulation [44, 45]. This region is extensively connected with limbic brain structures, such as the hippocampus, parahippocampal gyrus, amygdala, thalamus, cingulate and prefrontal cortices [46]. The involvement of the cerebellum (mainly the vermis subregion) in ED has been consistently demonstrated in several structural neuroimaging studies describing the presence of GM volume loss mainly in AN [47–49]. Moreover a recent resting state fMRI study [13], demonstrated the presence of altered intrinsic connectivity of the cerebellar vermis in both AN and BN patients. These authors hypothesized that this dysfunctional neural pattern might be related to some psychopathological aspects of ED (i.e., the drive thinness) that is pathologically altered in all ED patients. (b) The ACC, together with the OFC, are two regions taking part in the ventral limbic circuit, together with the amygdala, insula and ventral striatum, which are important for identifying the emotional significance of appetizing stimuli for inhibiting impulsive behaviors [14] and regulating reward systems [50]. The current neuroimaging literature mainly highlights the role of this neural network in pathophysiological mechanisms of BN, in which the alterations of mesolimbic reward response mechanisms could explain the lack of control and the impulsivity that are often present in BN patients and that are neurophysiologically expressed through dysfunctional activities in the ACC and OFC regions [15, 41]. However, fronto-striatal neural circuit dysfunctions related to altered reward processing have also described in AN patients [51], thus raising a different perspective in which stimuli that are otherwise aversive for healthy controls (e.g., self-starvation, emaciated body image) are considered rewarding and activate relevant reward linked brain regions in AN patients. (c) The involvement of the visual cortex is another key site associated with ED. Although altered functional activity of the occipital lobe has been reported in both AN and BN individuals [52], body image disturbance is fundamentally considered one of the core characteristics of AN. Several neuroimaging studies have described the neurobiological correlates of this symptom, defining the presence of a specific neural network involved in body processing: the fusiform area, the inferior temporal sulcus and the primary visual cortex. Recent evidence [53] demonstrated altered effective connectivity between these regions in AN patients during the viewing of bodies. (d) Finally, abnormal neural changes in the precuneus and sensorimotor/premotor cortices have been already described in both AN and BN patients [6, 13, 53]. Friederich et al. [41] showed that, using body images of slim fashion models to induce a self-other body shape comparison, AN patients had a higher activation of the premotor cortex. Again, Amianto et al., [13] found altered gray matter volume in the paracentral lobule, precuneus and somatosensory regions when comparing AN and BN patients, as well as the whole ED group, with respect to controls. Altered neural changes in brain areas involved in sensorimotor functions and visuo-proprioceptive information processing may either represent the physiological consequence of physical hyperactivity typical of ED patients [13] or as a dysfunction related to the body awareness. Body awareness is a complex cognition underpinned by aspects of visual perception, proprioception, and touch [54]. The processing of the body image concept requires integration of the different types of body-related perceptual experience and processing of information related to peripersonal space. The presence of altered anatomical changes in these regions together with visual cortex, has been interpreted as a dysfunctional processing of somatosensory information about the perceived body size [6, 55].

One important limitation of this study needs to be considered in discussion of our data: the clinical subtypes of the enrolled ED and the size of these groups. Considering a hypothetical structure of ED as a spectrum (in line with the trans-diagnostic approach of the DSM-V), in this study we enrolled the two extremes of the model. ED is not a uniform disorder characterized by a high heterogeneity in clinical phenotypes. For instance, the 60% of those who exhibit pathological ED behaviors but who do not meet the full criteria for AN or BN, are instead diagnosed as “eating Disorder Not Otherwise Specified” [56]. Again, the diagnosis is further complicated by the presence of other major psychiatric conditions [57], by disease duration [47] and severity of illness [58]. All this evidence highlights that our findings cannot be generalized to all ED populations. Moreover, the small sample size of AN patients as well as the fact that we only included outpatients with a lower disease duration and with mild severity of illness (BMI ~ 17) might have affected the magnitude of our classification Accuracy. Therefore, to sustain the usefulness of SVM application in clinical practice of ED, further studies are warranted employing a larger and heterogeneous sample. Despite this methodological limitation, it is important to highlight that the severe inclusion criteria employed in this study, albeit with a restricted sample selection, eliminated potential confounders (i.e., BMI), thus helping with the interpretation of the results.

In conclusion, our study demonstrates for the first time that using standard morphological brain images, SVM is able to extract neuroimaging biomarkers, which allow to accurately classify individuals with ED. Although we used this method in a diagnostic perspective, the rationale for applying machine learning methods in this psychiatric realm is to allow inferences to be made at the level of the individual for monitoring disease progression as well as improving prevention and treatment decisions. We believe that our preliminary findings offer new avenues for encouraging the application of these multivariate neuroimaging approaches in clinical practice, mainly to differentiate different ED phenotypes.

## Supplementary Material

In the Suppl Materials, we included additional clinical information about ED patients together with neuroimaging data related to voxel-based pattern distribution.

## Figures and Tables

**Table 1:** Demographic characteristics.

Variables	ED (*n* = 17)	HC (*n* = 17)	*P*-level
Demographical data			
Age (years)	30.2 ± 5.6	30.1 ± 5.5	0.95
Educational level (years)	17 (13–21)	17 (13–21)	0.88
BMI	23.6 ± 8.2	24.1 ± 4.8	0.79

MRI data			
Total GM Volume	587.3 ± 37.5	608.88 ± 42.1	0.11
Total WM Volume	486.5 ± 63.1	489.6 ± 41.6	0.86
Total CSF Volume	188.3 ± 28.7	187 ± 23.2	0.88

Clinical data			
HAMA	14.6 ± 13	4 ± 2.2	0.04^*∗*^
BDI	16.8 ± 10.1	6.3 ± 4.7	0.0004^*∗*^
DES	14.32 ± 12.4	5.12 ± 4	0.007^*∗*^
EAT-26	23.3 ± 14.4	6.35 ± 3.2	0.00004^*∗*^
SDQ-20	28.64 ± 14.8	20.6 ± 1.1	0.03^*∗*^
BIDA	29.9 ± 19.4	19.9 ± 11	0.24

Clinical data EDI-2 scale			
Drive for thinness	9.4 ± 6.3	1.2 ± 1.3	0.0001^*∗*^
Bulimia	3.47 ± 4.5	0.1 ± 0.5	0.01^*∗*^
Interoceptive awareness	7.9 ± 6.2	0.7 ± 1.2	0.0006^*∗*^
Asceticism	5.6 ± 3.8	2 ± 1.1	0.0006^*∗*^
Body dissatisfaction	12.9 ± 7.2	6.1 ± 2.9	0.001^*∗*^
Perfectionism	4.3 ± 3.9	3.3 ± 3.1	0.41
Interpersonal distrust	3.6 ± 3.1	1.4 ± 1.2	0.04^*∗*^
Impulse regulation	3.67 ± 4.9	0.6 ± 1.4	0.02^*∗*^
Ineffectiveness	3.5 ± 5.2	1.2 ± 2.6	0.12
Maturity fears	5.2 ± 3	3.94 ± 2.6	0.13
Social insecurity	3.53 ± 3.2	2.1 ± 2	0.22

Data are given as mean values (SD) or median values (range) when appropriate.

BMI: Body Mass Index; GM: gray matter; WM: white matter; CSF: cerebrospinal fluid; PBI: parental bonding instrument; STAI: State-Trait Anxiety Inventory; HAMA: Hamilton rating scale for anxiety; BDI: Beck Depression Inventory; DES: Dissociative Experiences Scale; EAT-26: eating attitude test-26; SDQ-20: Somatoform Dissociation Questionnaire-2; BIDA: Body Image Dimensional Assessment; EDI-2: Eating Disorder Inventory-2. Total brain MRI parameters have been calculated using VBM8 tool. ^*∗*^Significant difference.

**Table 2:** FDR values of the 30 features (PCA coefficients) used for the ED versus HC discrimination.

PCA coefficient (#)	FDR
1	0.2052
2	0.0172
3	0.0021
4	0.1286
5	0.0005
6	0.0786
7	0.1484
8	0.3923
9	0.0354
10	0.0137
11	0.0919
12	0.3376
13	0.1057
14	0.0002
15	0.0128
16	0.0176
17	0.0279
18	0.0188
19	0.0206
20	0.0511
21	0.0369
22	0.0001
23	0.0200
24	0.0052
25	0.1839
26	0.0431
27	0.0015
28	0.0250
29	0.0321
30	0.0171

Data from a single round of CV are shown as a representative example.

## Abbreviations

ED: Eating disorders
AN: Anorexia nervosa
BN: Bulimia nervosa
ACC: Anterior cingulate cortex
OFC: Orbitofrontal cortex
SVM: Support vector machine.
