# Supplementary material for: Biomarkers of Eating Disorders Using Support Vector Machine Analysis of Structural Neuroimaging Data: Preliminary Results
Source: Behav Neurol. 2015 Nov 18;2015:924814. doi: 10.1155/2015/924814 (PMC4663371; doi:10.1155/2015/924814)
Supplement: Supplementary file 1 — In the Suppl Materials, we included additional clinical information about ED patients together with neuroimaging data related to voxel-based pattern distribution. [file 924814.f1.pdf]

SUPPLEMENTARY MATERIALS

Biomarkers of eating disorders using support vector machine analysis of morphological imaging data: preliminary results.

Additional Clinical Data

To better characterize psychopathological profile of ED patients here we reported results obtained from additional clinical scales. ED patients were characterized by altered scores in PBI-Paternal Care (t= 6.42; p-level = 0.01) and PBI-Maternal Care (t= 15.8; p-level = 0.0003). Considering the presence of trauma, TEC revealed that only three healthy controls reported one traumatic experience (emotional neglect). In the ED group, four patients did not report trauma, while others patients reported more than one trauma. In particular, the 47% of the sample experienced emotional neglect, 41% emotional abuse, 53% sexual harassment, 23% sexual abuse and 29% physical abuse.

Finally, the MMPI-2 (Table S1) revealed several differences in the scores of clinical and content scales: Hypochondriasis (Hs) (t=15.1; p-level = 0.0005); Depression (D) (t=38.8; p-level = 0.00001); Hysteria (Hy) (t=23.6; p-level = 0.00003); Psychopathic deviate (Pd) (t=23.1; p-level = 0.0004); Paranoia (Pa) (t=12.5; p-level = 0.001); Psychastenia (Pt) (t=13.5; p-level = 0.0002); Schizoprenia (Sc) (t=12.6; p-level = 0.001); Hypomania (Ma) (t=8.1; p-level = 0.007); Social Introversion (SI) (t=4.5; p-level = 0.04); Anxiety (ANX) (t=10.7; p-level = 0.002); Obsessions (OBS) (t=6.1; p-level = 0.02); Depression (DEP) (t=17.1; p-level = 0.0002); Health concerns (HEA) (t=14.6; p-level = 0.0006); Family problems (FAM) (t=6.9; p-level = 0.01) and Work interference (WRK) (t=9.6; p-level = 0.008)

Table S1: MMPI-2 scores in eating disorders and control groups

Clinical Scales	ED	HC	*P-level*
L	55.2 ± 10.3	54.07 ± 7.2	0.53
F	58.2 ± 49.5	49.43 ± 8.4	0.06
K	49.5 ± 12.3	49.6 ± 40.4	0.18
**Hypochondriasis (Hs)**	**62.9 ± 14.8**	**50.87 ± 9.6**	**0.0005**
**Depression (D)**	**67.1 ± 10.5**	**47.3 ± 6.5**	**0.00001**
**Hysteria (Hy)**	**65.7 ± 13.1**	**46.8 ± 7.9**	**0.00003**
**Psychopathic deviate (Pd)**	**61.2 ± 6.6**	**47.6 ± 8.4**	**0.0004**
Masculinity-Femininity (Mf)	55.6 ± 9.7	50.1 ± 8.5	0.18
**Paranoia (Pa)**	**58.5 ± 12.7**	**48.73 ± 9.1**	**0.001**
**Psychastenia (Pt)**	**56.2 ± 7.5**	**45.5 ± 6.9**	**0.0002**
**Schizophrenia (Sc)**	**57.1 ± 12.6**	**44.37 ± 7.5**	**0.001**
**Hypomania (Ma)**	**55.2 ± 9.4**	**45 ± 7.6**	**0.007**
**Social Introversion (SI)**	**52.7 ± 13.8**	**45.4 ± 7.8**	**0.04**
**Content Scales**	**ED**	**HC**	***P-level***
**Anxiety (ANX)**	**59.7 ± 13.9**	**45.5 ± 8.9**	**0.002**
Fears (FRS)	48.5 ± 11.5	46.6 ± 8.4	0.52
**Obsessions (OBS)**	**55.1 ± 13.4**	**45.7 ± 9.8**	**0.02**
**Depression (DEP)**	**61.2 ± 13.1**	**44.4 ± 10.7**	**0.0002**
**Health concerns (HEA)**	**63.5 ± 15.1**	**46.1 ± 8.7**	**0.0006**
Bizarre Mentation (BIZ)	49.7 ± 12.6	47.1 ± 7.9	0.36
Anger (ANG)	54 ± 13.7	48.6 ± 10.3	0.44
Cynicism (CYN)	49 ± 11.3	49.5 ± 10.2	0.75
Antisocial practises (ASP)	46.4 ± 9.6	45.57 ± 8.2	0.31
Type A (TPA)	46.9 ± 10.3	45.5 ± 9.7	0.73
Low self-esteem (LES)	50 ± 12.8	45.5 ± 10.5	0.06
Social discomfort (SOD)	49 ± 9.7	43.3 ± 7.9	0.08
**Family problems (FAM)**	**54.1 ± 10.7**	**44.8 ± 7.8**	**0.01**
**Work interference (WRK)**	**55.3 ± 13.7**	**44.9 ± 8.5**	**0.008**
Negative treatment indicators (TRT)	52.5 ± 17.9	43.6 ± 9.2	0.06

Voxel-based pattern distribution

Figure S1 shows the SVM weight map displaying the weight (normalized to a range between 0 and 1) of each voxel assigned by SVM during the training phase for the classification between ED patients and HC. Higher weights are localized in the primary visual cortex, cerebellar vermis and tonsil together with the ACC.

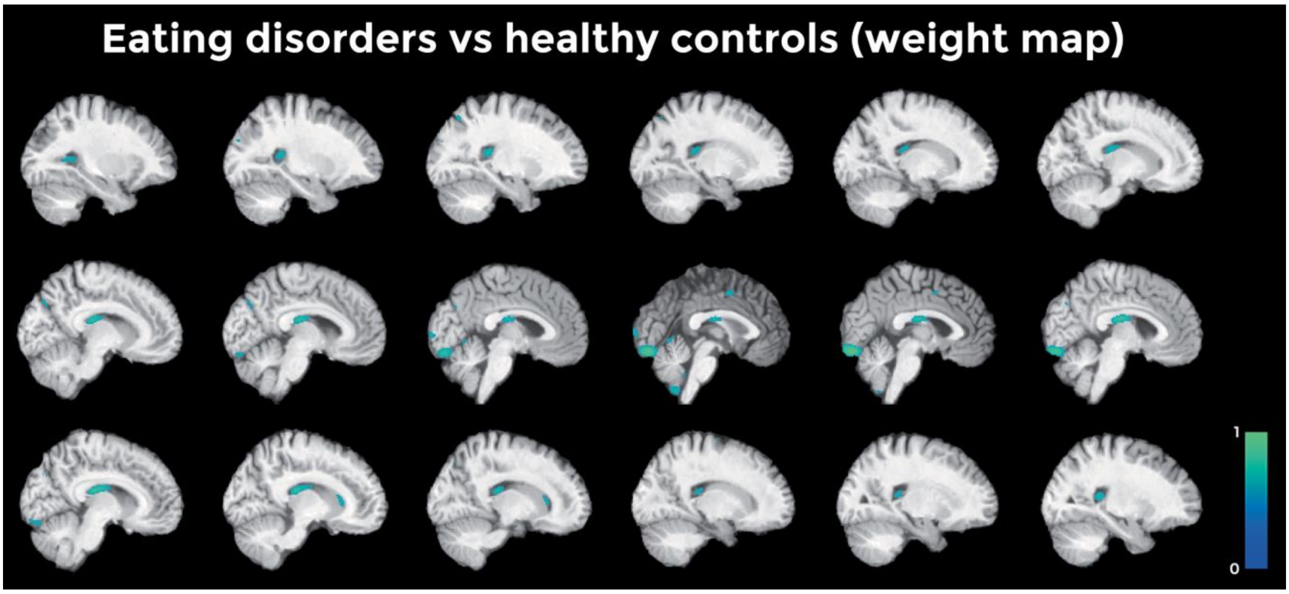

Figure S1: *Weight map for the classification between ED patients and healthy controls (sagittal view, threshold = 60%). These maps show the weight (normalized to a range between 0 and 1) assigned by SVM during the training phase to each voxel for the discrimination between ED and HC. Voxel-based weight distribution is expressed according to the color scale and superimposed on a standard stereotactic brain for spatial localization.*
